# A Feedback Loop between TGF-β1 and *ATG5* Mediated by miR-122-5p Regulates Fibrosis and EMT in Human Trabecular Meshwork Cells

**DOI:** 10.3390/cimb45030154

**Published:** 2023-03-13

**Authors:** Munmun Chakraborthy, Aparna Rao

**Affiliations:** 1 Hyderabad Eye Research Foundation (HERF), L.V. Prasad Eye Institute, Bhubaneswar 751024, Odisha, India; 2 School of Biotechnology, KIIT University, Bhubaneswar 751024, Odisha, India

**Keywords:** TGF-β1, *ATG5*, miR-122-5p, feedback loop

## Abstract

Autophagy is a cell’s evolutionary conserved process for degrading and recycling cellular proteins and removing damaged organelles. There has been an increasing interest in identifying the basic cellular mechanism of autophagy and its implications in health and illness during the last decade. Many proteinopathies such as Alzheimer’s and Huntington’s disease are reported to be associated with impaired autophagy. The functional significance of autophagy in exfoliation syndrome/exfoliation glaucoma (XFS/XFG), remains unknown though it is presumed to be impaired autophagy to be responsible for the aggregopathy characteristic of this disease. In the current study we have shown that autophagy or ATG5 is enhanced in response to TGF-β1 in human trabecular meshwork (HTM) cells and TGF-β1 induced autophagy is necessary for increased expression of profibrotic proteins and epithelial to mesenchymal (EMT) through Smad3 that lead to aggregopathy. Inhibition of *ATG5* by siRNA mediated knockdown reduced profibrotic and EMT markers and increased protein aggregates in the presence of TGF-β1 stimulation. The miR-122-5p, which was increased upon TGF exposure, was also reduced upon *ATG5* inhibition. We thus conclude that TGF-β1 induces autophagy in primary HTM cells and a positive feedback loop exists between TGF-β1 and *ATG5* that regulated TGF downstream effects mainly mediated by Smad3 signaling with miR-122-5p also playing a role.

## 1. Introduction

Pseudoexfoliation syndrome (XFS) is a protein aggregation disorder and the most common cause of exfoliation glaucoma (XFG). XFS is characterized by white flaky deposits seen over ocular surfaces. Transforming growth factor-beta1 (TGF-β1) which has been shown to be increased in XFS and XFG [1,2], not only plays a specialized role in the body, it also regulates other pathways, which helps to explain why TGF-signalling dysregulation is linked to a variety of disorders. TGF also regulates extracellular matrix homeostasis, which is known to be dysregulated in glaucoma, in XFS/XFG [1,2]. Abnormal extracellular matrix (ECM) production and reduced clearance of ECM aggregates are known to be caused by TGF in several fibroproliferative diseases, including XFG [1,3,4]. In XFG, the accumulation of protein complex aggregates in the trabecular meshwork causes a blockage of the outflow channels leading to raised intraocular pressure, glaucoma, and subsequent blindness. The trabecular meshwork, therefore, constitutes the tissue of end-organ damage in glaucoma and XFG, which therefore is the primary site of molecular events preceding cell death or dysfunction. In several studies [3,4,5], the role of dysregulated autophagy and TGF-β signaling pathways in the pathogenesis of kidney fibrosis, diabetes, and its complications, such as cardiomyopathy, retinopathy, and nephropathy, has been reported. In our previous study, we have also shown that 10 ng/mL TGF-β1 causes an increase in ECM protein production, causes apoptosis, induces epithelial to mesenchymal transition (EMT), and protein aggregation in HTM cells in vitro [6]. The reduction of cellular degradative processes, particularly the autophagy–lysosomal pathway, is linked to protein aggregation. We recently discovered that in all stages of XFG, lower unfolded protein response (UPR) clearance is related to elevated TGF levels, implying that the autophagy pathway and TGF-β autophagy crosstalk may be involved in aggregate clearance [1,7]. Autophagy is an intracellular trafficking system that transports cytosolic elements to the lysosome for destruction, which is necessary for misfolded protein clearance, ubiquitin–proteasomal degradation, and cell repair [7,8,9,10,11,12]. Autophagy is a protective mechanism that can be triggered by a variety of intracellular and extracellular stimuli, including a lack of amino acids or growth hormones, hypoxia, a low cellular energy status, endoplasmic reticulum stress or oxidative stress, organelle injury, and pathogen infection [5,7,13,14,15,16,17,18,19]. While autophagy is a driving force in the regulation of cell viability and function, it is also regarded as the second kind of programmed cell death, with autophagosome accumulation differing from apoptosis [20,21]. Micro ribonucleic acids (MicroRNAs), which are noncoding RNAs, have also been shown to regulate autophagy either directly or through a variety of routes, and so have an important role in the cause and treatment of a variety of disorders. Given the role of autophagic clearance of protein aggregates, autophagy-related genes (*ATG* genes) may be involved in XFG pathophysiology. Furthermore, the potential for autophagy and TGF to interact has attracted a lot of attention. The general consensus is that autophagic pathways are beneficial for cell functions since they facilitate the elimination of aggregates that are too big for proteasome-mediated clearance. In fact, there is a clear link reported between autophagy induction and the existence of protein aggregates in various neurodegenerative disorders, including Alzheimer’s disease [22], Huntington’s disease [23], and amyotrophic lateral sclerosis [24]. In light of this evidence, it would be interesting to look into the role of autophagy and TGF signaling pathways, as well as their crosstalk, in the progression of XFS/XFG, in order to spur the development of targeted therapeutic strategies. We have reported that a feedback loop exists between TGF-β1 and *ATG5* which regulates the induction of profibrotic proteins and EMT. Further research into autophagy in XFS and XFG could lead to new knowledge of the disease’s pathogenesis as well as new therapeutic options in the future.

## 2. Materials and Methods

### 2.1. siRNA Transfection

The siRNA transfection was carried out as previously described [25]. Briefly, 60 pmol/well of siRNAs against autophagy-essential ATG5 (siATG5) (6345S, Cell signalling technology, Beverly, MA, USA)and nontargeting siRNA (negative control, siNC) (6568S, Cell signalling technology, Beverly, MA, USA) in serum-free medium was mixed with Lipofectamine RNAiMAX reagent (13778, Invitrogen, Beverly, MA, USA) and incubated for 60 min according to the instructions provided by the manufacturers. Passage 4 HTM cells were then suspended in the siRNA–lipofectamine mixture and plated on 6 well plates(reverse transfection approach for successful siRNA distribution). Cells were then cultivated and treated accordingly and used for further analysis. Transient knockdown of the ATG5 gene was chosen because ATG5 participates in both for autophagy induction and conjugation cascades leading to microtubule-associated protein light chain (LC3) lipidation and autophagosome formation [12], thus silencing autophagy.

### 2.2. Stimulation of HTM Cells with TGF-β1

We isolated primary HTM cells from healthy cadaver eyes procured from the institutional eye bank (age of donors 28 ± 1.2 years, all cadaver corneas procured within 4 h of death). The use of human tissue in this study adhered to the tenets of the Declaration of Helsinki. Briefly, the trabecular meshwork (TM) stripped from the procured corneoscleral rims were thoroughly washed with Dulbecco’s-phosphate buffer saline (D-PBS, A12856-01, Gibco, Carlsbad, CA, USA) followed by dissection of the TM tissue into tiny pieces with microscissors under a dissection microscope (Olympus SZX7, Tokyo, Japan). The cut TM pieces were digested with collagenase treatment (1 mg/mL at 37 °C for 2 h) which was followed by centrifugation (1200 rpm, 5 min) and trypsinization (0.25% trypsin, 5 min). The TM cells were pelleted down by centrifugation (1200 rpm, 5 min) and plated on a 35 mm dish with an endothelial cell basal medium, EBM-2 media (cc-3156, Lonza, Basel, Switzerland) at 37 °C and 5% CO_2_. Primary HTM cells (passage 1 to 5) infected with and without siRNA (siATG5 and siNC) were cultured to 80% confluency in Dulbecco’s modified eagle medium (DMEM) + 10% fetal bovine serum (FBS). Cells were then serum-starved DMEM (0% FBS) for 24 h before being treated with TGF-β1 (10 ng/mL) for 0–72 h in preparation for the experiment.

### 2.3. RNA Isolation and Quantitative Polymerase Chain Reaction (qPCR)

QIAzol lysis reagent was used to extract total RNA from control and treated cells (passage 3) (QIAGEN, Hilden, Germany). One µg of RNA was reverse-transcribed using the Reverse Transcriptase Core Kit (RT-RTCK-03, EUROGENTEC, Liege, Belgium), and the cDNA was subjected to quantitative PCR using the PowerUp SYBR Green qPCR Master Mix(A25741, Applied Biosystems, Foster City, CA, USA) with the following reaction conditions: Stage 1: 50 °C for 2 min, Stage 2 (40 cycles): 94 °C for 2 min, 95 °C for 3 s and 60 °C for 30 s (for Primers: ATG5 Forward-TGGGATTGCTCAGGCAACGAA, Reverse-TTCCCCATCTTCAGGATCAA and LC3-II Forward-GAGAAGCTTCCTGTTCTGG, Reverse-GTGTCCGTTCACCAACAGGAAG). The data were examined using the threshold (Ct) method after the Ct values were standardized to GAPDH, which served as an internal reference.

We also used the Applied Biosystems TaqMan Advanced miRNA Assay kit (A25576, Applied Biosystems, Foster City, CA, USA) which detects and quantifies mature miRNA from as little as 1 pg of total RNA. After RNA preparation, cDNA was synthesized using the TaqMan Advanced miRNA cDNA Synthesis Kit. The cDNA is then preamplified using universal primers and a master mix to uniformly increase the amount of cDNA for each target, maintaining the relative differential levels after which the expression levels of miR-122-5p, miR-124-3p, and miR-424-5p) are quantified. The Ct values were standardized to U6 (RNU6-1), which served as an internal reference.

### 2.4. Annexin V/propidium Iodide (PI) Staining Assay

Apoptosis was examined in TGF-β1 and siRNA-treated and untreated cultures (passage 3) using the annexin V-fluorescein isothiocyanate (FITC)/prodium iodide (PI) dual staining method with the BD FACS Canto II Flow Cytometer (BD Biosciences, San Jose, CA, USA) using the Annexin V-FITC Apoptosis Detection kit (MintenyiBiotec, Bergisch, Gladbach, Germany). The percentage of the total (early + late) apoptotic cells were plotted.

### 2.5. Protein Whole Cell Lysate and Immunoblotting

Immunoblotting was performed using a method previously described [6]. Briefly, cells (passage 4) were treated accordingly, and protein lysates were extracted in RIPA buffer containing protease inhibitors, 2.0 mM N-ethylmaleimide, 2.0 mM 4-(2-aminoethyl)-benzenesulfonyl fluoride, 0.05 M Tris-HCl (pH 8.0), 0.15 M sodium chloride (NaCl), 5.0 mM ethylenediaminetetraacetic acid (EDTA), and 1% NP-40. The Bradford assay was used to quantify the protein, and an equal amount of protein was placed onto each lane of 10–12% polyacrylamide SDS-PAGE gels and transferred to polyvinylidene difluoride (PVDF) membranes (Merck Millipore). Membranes were blocked with 5% skimmed milk and incubated with antibodies. The primary antibodies used in the study were mouse anti-SMA (ab7817, 1:1000, Abcam, Cambridge, MA, USA), antivimentin (D21H3, 1:1000, Cell Signalling Technology, Beverly, MA, USA), rabbit antifibronectin (AF5335, 1:1000, Affinity Biosciences, Brisbane, QLD, Australia), mouse anti-fibrillin (AF0429, 1:1000, Affinity Biosciences, Brisbane, QLD, Australia), rabbit anti-ATG-5 (DF6010, 1:800, Affinity Biosciences, Brisbane, QLD, Australia), anti-LC3I/II (AF5402, 1:1000, Affinity Biosciences, Brisbane, QLD, Australia), and SMAD3 (MA5-14939, Abcam, Cambridge, MA, USA). The loading control used was rabbit anti-GAPDH (1:10,000; Abcam, Cambridge, MA, USA).

The primary HTM cells were treated with Smad3 inhibitor, SIS3 (CAS 1009104-85-1), with and without TGF-β1 treatment and the protein levels of ATG-5, LC3-I and LC3-II were validated.

### 2.6. Electron Micrograph and Immunofluorescence Staining

Cells (passage 5) were treated with TGF-β1 for 72h, pelleted down, and fixed in 2% glutaraldehyde in 0.1 mol/L phosphate buffer for 15 h, then postfixed in 2% buffered osmium tetroxide and embedded in epoxy resin (Epon) in a usual method. Ultrathin sections stained with uranyl acetate–lead citrate mixture were then seen under an electron microscope (JEOL, JEM-2100 PLUS, Akishima, Tokyo, Japan).

For immunostaining, control and siATG5 transfected cells were sown in coverslips covered with 0.1% gelatin before TGF-β1 treatment. The cell culture medium was separated and the cells were rinsed in phosphate buffer saline (PBS) before being fixed for 15 min at room temperature (RT) with 4% paraformaldehyde. The cells were then permeabilized with a 0.2% Triton X-100 solution for 10 min before being incubated with a blocking solution (5% FBS in 1X PBS) for 20 min at RT with gentle rocking to block nonspecific sites. Following this, the cells were incubated overnight at 4 °C with primary antibodies, Oligomer 11 (AHB0052, 1:1500, Invitrogen, Beverly, MA, USA) and amyloid fibril (PA5-77843, 1:1500, Invitrogen, Beverly, MA, USA), followed by 1 h with secondary antibody (antirabbit Alexa 488, 1:2000, Invitrogen, Beverly, MA, USA) mixed with 2 g/mL Hoechst (four nucleus staining) at RT. The coverslips were mounted and examined under a fluorescence microscope (Olympus, BX53, Tokyo, Japan).

### 2.7. Statistical Analysis

Each experiment was carried out three times. The mean data were reported as mean +/− standard deviation. Graphpad prism (Version 7, San Diego, CA, USA) was used for statistical analysis. An unpaired Student *t*-test was performed to determine statistical differences between the two groups, and a one-way ANOVA with post hoc analysis was employed for >2 groups, with *p* < 0.05 set as statistical significance.

## 3. Results

### 3.1. TGF-β1 Induced ATG5 Activation, Fibrosis, and Epithelial to Mesenchymal Transition in HTM

**Figure 1:**
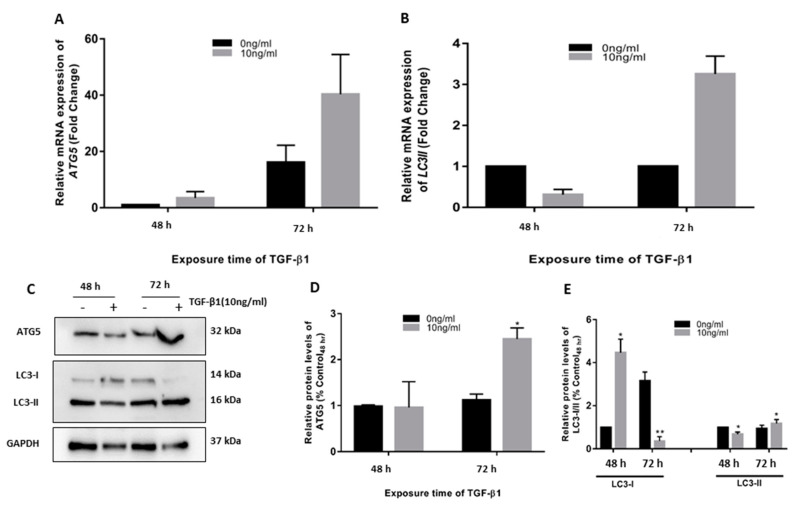
TGF-β1 induced ATG5 activation in human trabecular meshwork (HTM cells). TGF-β1 (10 ng/mL) applied to primary HTM cells for 48–72 h, increased relative mRNA expression of (**A**) *ATG-5* and (**B**) LC3-II. It also increased levels of (**C**) ATG-5 proteins and lipidation of LC3 II, (**D**,**E**) Densitometry analysis of bands. The results represent the averages of three separate tests conducted. The error bars represent the standard deviation (*—*p* < 0.05, **—*p* < 0.01, by *t*-test).

TGF-β1 is one of a number of factors involved in fibrogenesis and EMT. Our group has previously studied and reported TGF-β1-induced fibrosis and EMT in HTM cells [6]. Our first objective was to see if TGF-β1 also induces autophagy in HTM cells. We observed that 10 ng/mL TGF-β1 caused considerable increases in autophagy markers, ATG5, and LC3-II at 72 h as compared to time-matched controls. A significant increase in protein levels of ATG5 and LC-I and II was also found (Figure 1).

### 3.2. TGF-β1-Induced Fibrosis and Epithelial to Mesenchymal Transition Is Regulated by Autophagy Induction

**Figure 2:**
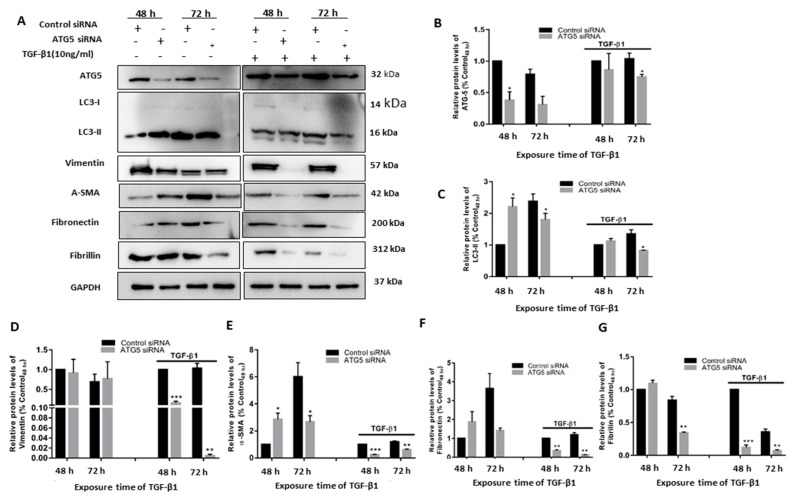
TGF-induced fibrosis and EMT is regulated by ATG5. (**A**) The protein levels of ATG-5, LC3-I, LC3-II, SMA, vimentin, fibronectin, and fibrillin in whole-cell lysates of siRNA-mediated knockdown HTM cells with (10 ng/mL) and without TGF-β1. (**B**–**G**), relative quantification of the proteins obtained using densitometry analysis of the bands. The error bars represent the standard deviation (*—*p* < 0.05, **—*p* < 0.01, ***—*p* < 0.001, by *t*-test).

We investigated the effects of TGF-β1 activation of HTM in the presence of autophagy inhibition to see if the link between autophagy and TGF-β1-induced fibrosis was more than correlational. We employed siRNA-mediated transient *ATG5* inhibition (si*ATG5*) to curtail autophagy (autophagy inhibition was confirmed by reduced ATG5 protein levels) and analyzed the existence of autophagy flux to get a more quantitative assessment of autophagy induction. Inhibition of the autophagy gene *ATG5* dramatically reduced the α-SMA and vimentin increase induced by TGF-β1. We also investigated the levels of fibronectin and fibrillin to see if this inhibitory impact was also relevant to the profibrotic proteins. Silencing *ATG5* also reduced the TGF-induced expression of fibronectin and fibrillin, as demonstrated in Figure 2. The accumulation of LC3-II caused by TGF-β1 was increased in the si*ATG5* transfected cells. These results in summary support our hypothesis that TGF-β1 regulates and induces EMT and fibrosis in HTM cells via crosstalk with the autophagy pathway.

### 3.3. TGF-Autophagy Crosstalk Regulates Apoptosis in HTM Cells

**Figure 3:**
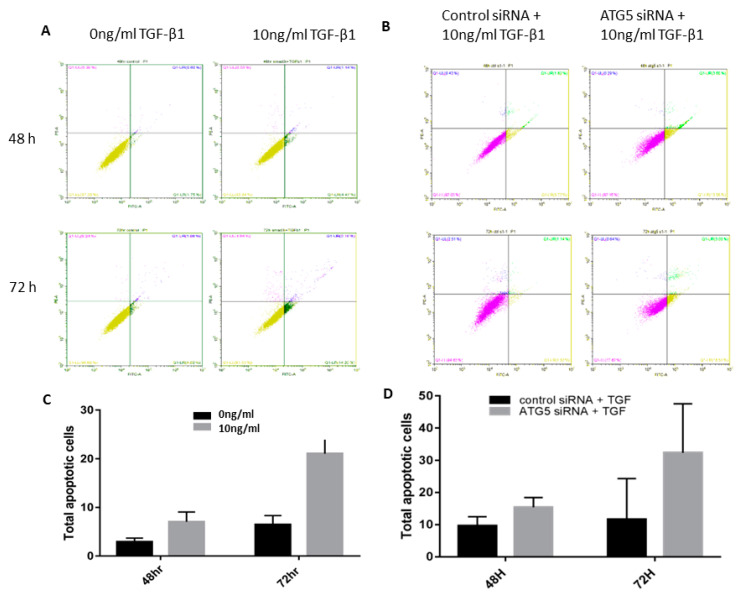
Apoptosis is regulated by ATG5 in HTM cells. HTM cells were treated with TGF-β1 (10 ng/mL) and siRNA and the total number of apoptotic cells was calculated by flow cytometry. (**A**–**D**) The total number of apoptotic cells in TGF-β1 treated and siRNA knockdown HTM cells.

Autophagy and apoptosis have been previously demonstrated to either regulate each other or occur independently. Our data showed that 10 ng/mL TGF-β1 activates both autophagy and apoptosis and autophagy inhibition increased apoptosis suggesting that apoptosis is regulated by ATG5 in HTM cells and inhibition of autophagy causes apoptosis in HTM cells (Figure 3).

### 3.4. TGF Induced Autophagy Involves a Positive Feedback Loop of ATG5 on the TGF Pathway

**Figure 4:**
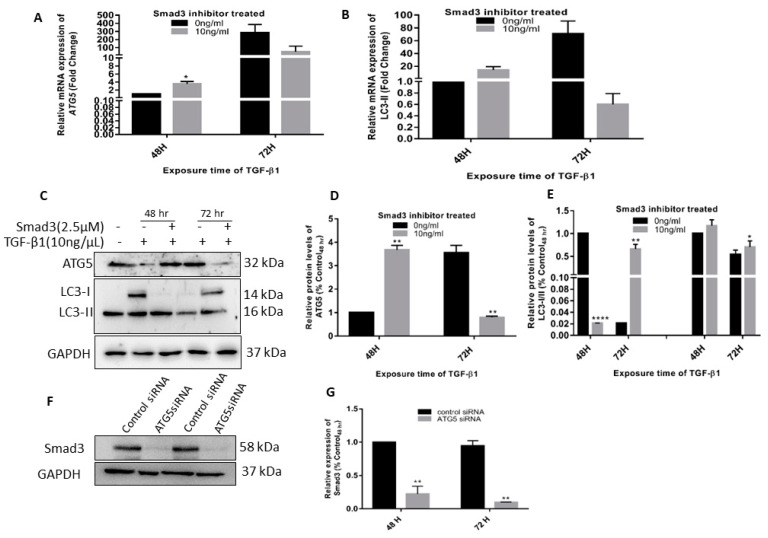
*ATG5* regulates TGF-β1 induced effects in HTM cells via Smad3 signaling. HTM primary cells treated with 2.5 µM Smad3 inhibitor (SIS3) and with (10 ng/mL) or without TGF-β1 showed a relatively lower expression of autophagy markers, (**A**) ATG-5, and (**B**) LC3-II as compared to time-matched controls. (**C**) Western blot analysis of ATG-5 and LC3-I/II proteins shows a similar trend, (**D**,**E**) densitometry analysis of the bands, (**F**) Levels of Smad3 in ATG5 inhibited cells and (**G**) densitometry analysis of Smad3 bands. The error bars represent standard deviation (*—*p* < 0.05, **—*p* < 0.01, ****—*p* < 0.0001, by *t*-test).

As shown in Figure 4, Smad3 inhibitor-treated cells had lower levels of autophagy proteins ATG5 and LC3-II. This also paralleled with reduced Smad3 protein levels in si*ATG5* transfected cells confirming that ATG5 regulates TGF-β1 induced EMT and fibrosis in HTM cells via a positive feedback loop of ATG5 involving TGF-β1 and SMAD signaling and downstream effects.

### 3.5. Crosstalk between ATG5 and TGF May Be Mediated by miR 122-5p

**Figure 5:**
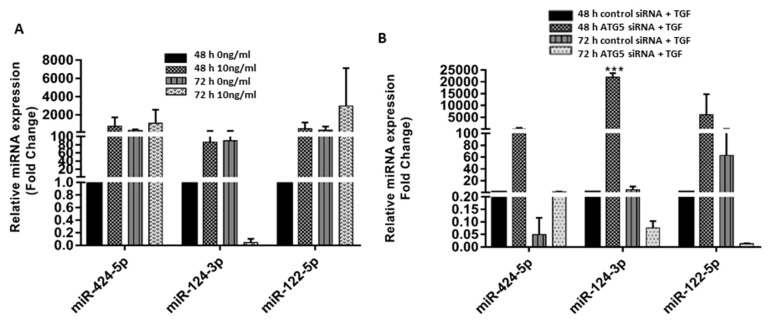
ATG5-TGF crosstalk may be mediated by miR-122-5p. Relative miRNA expression levels of, miR-424-5p, miR-124-3p, and miR-122-5p in (**A**) Transforming growth factor, TGF-β1 (10 ng/mL) treated human trabecular meshwork (HTM) cells and (**B**) siRNA mediated knockdown HTM cells with (10 ng/mL) and without TGF-β1 exposure. The error bars represent standard deviation ***—*p* < 0.001, by one-way ANOVA).

In our previous study [1], we found three novel miRNAs (miR-122-5p, miR-124-3p, and miR-424-5p) that were involved in pathways, namely TGF-β1, fibrosis/ECM, and proteoglycan metabolism. Since ATG5 was found to regulate TGF-β1 induced EMT and fibrosis through the Smad3 pathway, we evaluated the expression of these three miRNAs in TGF-β1 treated and siATG5-inhibited HTM cells to see if the TGF and autophagy crosstalk involves miRNAs. We observed an increase in the expression of miR-122-5p and a decrease in miR-124-3p at 72 h in TGF-β1 stimulated cells (Figure 5A). This paralleled with a decrease in the expression of miR-122-5p at 72 h in si*ATG5* cells stimulated with TGF-β1 (Figure 5B) indicating the possibility that ATG5 could be modulating miR-122-5p which in turn modulates Smad3 signaling in HTM cells. This, however, requires further validation.

### 3.6. TGF-Induced ATG5 Activation Is Essential to Prevent TGF-Induced Aggregate Formation in HTM Cells

**Figure 6:**
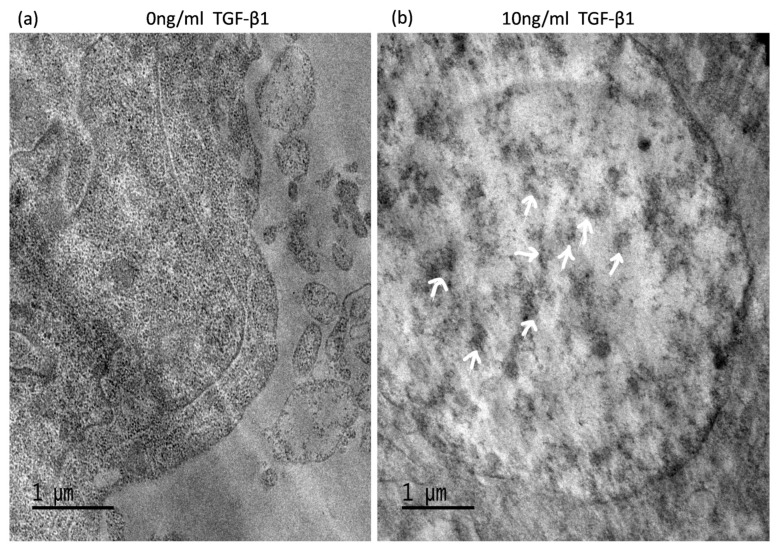
Electron microscopic appearance of protein aggregates. Control (**a**) and 10 ng/mL TGF-β1 treated cells (**b**) were observed under electron microscope. One-µm thin slices show protein aggregate localization (indicated by white arrows, (**b**) in 72 h, 10 ng/mL TGF-β1 treated cells (original magnification ×50,000).

**Figure 7:**
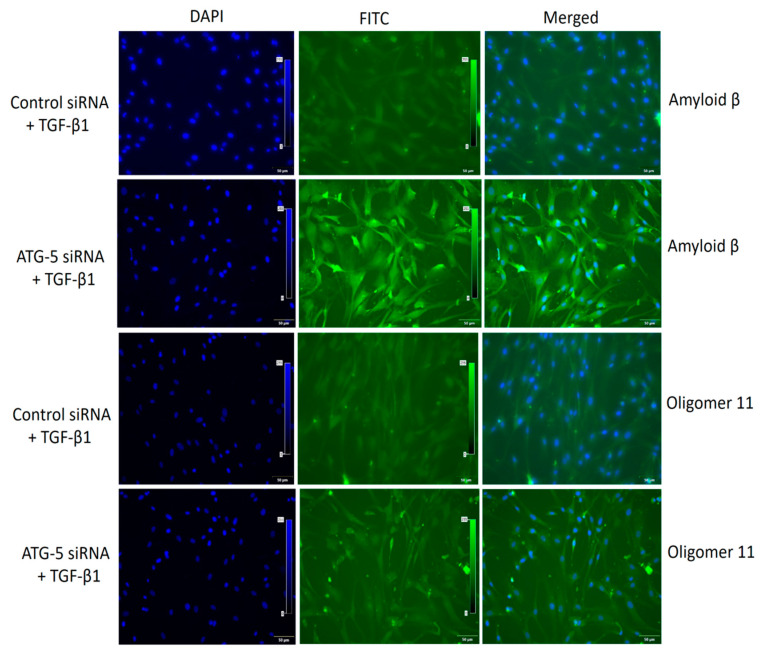
Transforming growth factor, TGF induced ATG5 activation plays a crucial role in preventing TGF induced aggregate formation in HTM cells. Amyloid fibrils were found around and closer to the nucleus and oligomer 11 was spread throughout in siATG5 cells exposed to 10 ng/mL TGF-β1. Magnification 20×, Bar = 50 µM.

Electron micrograph results confirmed the presence of protein aggregates occurring in 72h TGF-β1 treated cells (Figure 6). Immunofluorescence images of si*ATG5* HTM cells treated with TGF-β1 at 10 ng/mL for 72 h showed aggregate deposition, suggesting the role of autophagy in protein aggregation. Amyloid fibrills were restricted near the nucleus and oligomer 11 was spread throughout the cell and ECM (Figure 7). ATG-5 inhibition however resulted in increased protein aggregation implying that autophagy activation is crucial for aggregate clearance. The results also suggest that TGF possibly mitigates aggregate formation independently and clearance requires autophagy activation.

## 4. Discussion

In this investigation, we found that TGF-β1 increases autophagy and the siRNA-mediated restriction of *ATG5* led to a positive feedback loop repressing TGF-induced fibrosis and EMT. This feedback loop between ATG5 and TGF-β1 is possibly mediated via miR-122-5p and Smad3 pathway, which may be a therapeutic target to reduce EMT, fibrosis, and prevent or reduce aggregate formation in XFS.

Autophagy is a biological process that provides a system for protein breakdown, which is necessary for tissue regeneration, cell survival, and homeostasis. Several studies have reported the crosstalk between autophagy and TGF-β1 in various diseases. TGF-induced fibrosis and EMT have been well studied in the pathophysiology of XFS and XFG [2,6]. Literature has recently focused on the interaction between autophagy, TGF, and the fibrotic response in various degenerative diseases [25,26,27,28,29,30,31]. Autophagy can either positively or negatively control fibrosis, depending on the cell type, tissue, or pathological circumstances. We found in this study that suppressing autophagy partially dramatically reduces TGF-β1-mediated Smad signaling and downstream effects on profibrotic factors in HTM cells. Furthermore, in si*ATG5*-transfected cultures, the vimentin and SMA levels in response to exogenous TGF-β1 therapy were significantly reduced. Fibrosis has been linked to both autophagic upregulation and downregulation in many organs, highlighting the diversity of autophagy’s functional involvement in tissue repair. Consistent with the findings of our study, another study has reported that TGF-β1 promotes both COL1A2 synthesis and autophagy induction in human atrial myofibroblasts. Knocking out *ATG5* in mouse embryonic fibroblasts also has been reported to result in a decrease in TGF-β1-induced fibrosis, when compared to wild-type cells, emphasizing the importance of autophagy in TGF-β1 induced fibrosis [32]. Interestingly, autophagy inhibition in human and murine macrophages resulted in a greater reduction in TGFB1 production [33]. Likewise, another group showed that the reduction in TGF-induced fibrosis was linked to genetic and pharmacological suppression of autophagy, which was generated by a BAMBI-mediated lower activation of Smad2/3 signaling in autophagy-deficient cells [34]. A feedback loop between ATG5 and TGF, as shown in this study, is a protective mechanism to control TGF-mediated side effects like EMT, cell death, and fibrosis, that may be detrimental in vivo in the HTM. Our earlier study showed that continued expression of TGF causes dysregulation of the downstream effects with enhanced cell death and preferential upregulation of specific profibrotic markers and miRNAs. While TGF is directly known to regulate ATG5 and autophagosome functioning, [3,25,26], this study suggests a dysfunctional feedback loop and aberrant crosstalk of TGF with ATG5, which may explain some of the pathogenic features seen in PXF.

Eyes with XFS have flaky protein aggregate deposits that have been associated with increased TGF-β1 expression in XFS/XFG [1,2,7]. An in vitro model that involved costimulating retinal pigment epithelial (RPE) cell monolayers with TNF-α and TGF-β2 resulted in the formation of cellular aggregates [35]. Mutant TGF-β1was shown to enhance Aβ peptide aggregation in corneal dystrophy, suggesting the potential direct role of TGF in mediating aggregate formation [36]. In our previous study, we reported protein aggregates in HTM cells upon continued TGF-β1 exposure. We also showed a decreased UPR response in patients with pseudoexfoliation glaucoma, suggesting that reduced autophagy-induced UPR regulation was key in the formation of aggregates in the trabecular meshwork in advanced PXF disease. Therapeutic strategies targeting the formation or dissolution of aggregates in XFS/XFG may involve targets that mediate these feedback loops between TGF, ATG5, and the UPR pathway.

A growing body of evidence suggesting the function of hsmiR-122-5p in downregulating the TGF–Smad pathway has been established. Further, miR-122 plays a role in skeletal muscle myogenesis by regulating the TGF–Smad pathway [37]. MicroRNA and miR-122-5p were found to be downregulated in skeletal muscle fibrosis as a result of TGF [38]. Findings of one study revealed that the miR-122–PKM2 autophagy axis protects hepatocytes against arsenite stress via the PI3K–Akt–mTOR pathway, suggesting that miR-122 could be a possibility for arseniasis treatment [39]. This study shows miR-122-5p upregulation upon TGF response that was downregulated in si*ATG5* knockdown cells, indicating the possibility that *ATG5* feedback loop regulates TGF/Smad signaling via miR-122-5p.

Our findings highlight the importance of autophagy and its link to the TGF pathway in the processes of protein aggregates in HTM cells. They also provide a new paradigm for understanding the causes of fibrosis and HTM cell dysfunction that are key to understanding the pathophysiology of glaucoma in XFS. Overall, the findings strongly suggest that autophagy and TGF play key balancing roles in HTM cell function and regulation of fibrotic response. Finally, these findings point to autophagy and aggregated proteins as potential new therapeutic targets for reversing or preventing HTM cell death in XFG.

## Data Availability

Not applicable.
